# Revolutionizing Rehabilitation: The Legacy of Dr. Pramod Karan Sethi and the Jaipur Foot

**DOI:** 10.7759/cureus.69493

**Published:** 2024-09-15

**Authors:** Mitul Saha, Sonali G Choudhari

**Affiliations:** 1 Department of Community Medicine, Jawaharlal Nehru Medical College, School of Epidemiology and Public Health, Datta Meghe Institute of Higher Education & Research, Wardha, IND

**Keywords:** amputee care, "historical vignette", jaipur foot, padma shri, prosthetic, ramon magsaysay award, rehabilitation

## Abstract

Dr. Pramod Karan Sethi was a surgery lecturer who created the Orthopaedic Department and Rehabilitation Unit at the Sawai Man Singh Medical College and Hospital, Jaipur. He paired with a local craftsman, Pandit Ram Chandra Sharma, to create the well-known "Jaipur Foot". This affordable and durable prosthetic foot is aimed to meet the needs of amputees in both developing and developed countries. It allowed users to sit cross-legged, squat, and walk on rough ground. The invention of the Jaipur Foot also had a big impact on Sudha Chandran, an Indian actress and Bharatanatyam dancer’s life. This feature was absent in the inflexible Western prosthetics available at that time. This biography explores Dr. Sethis's education and career path. It also looks into the challenges he faced while developing the Jaipur Foot. It also discusses his contribution to polio victim rehabilitation and his influence on amputee care worldwide.

## Introduction and background

**Figure 1:**
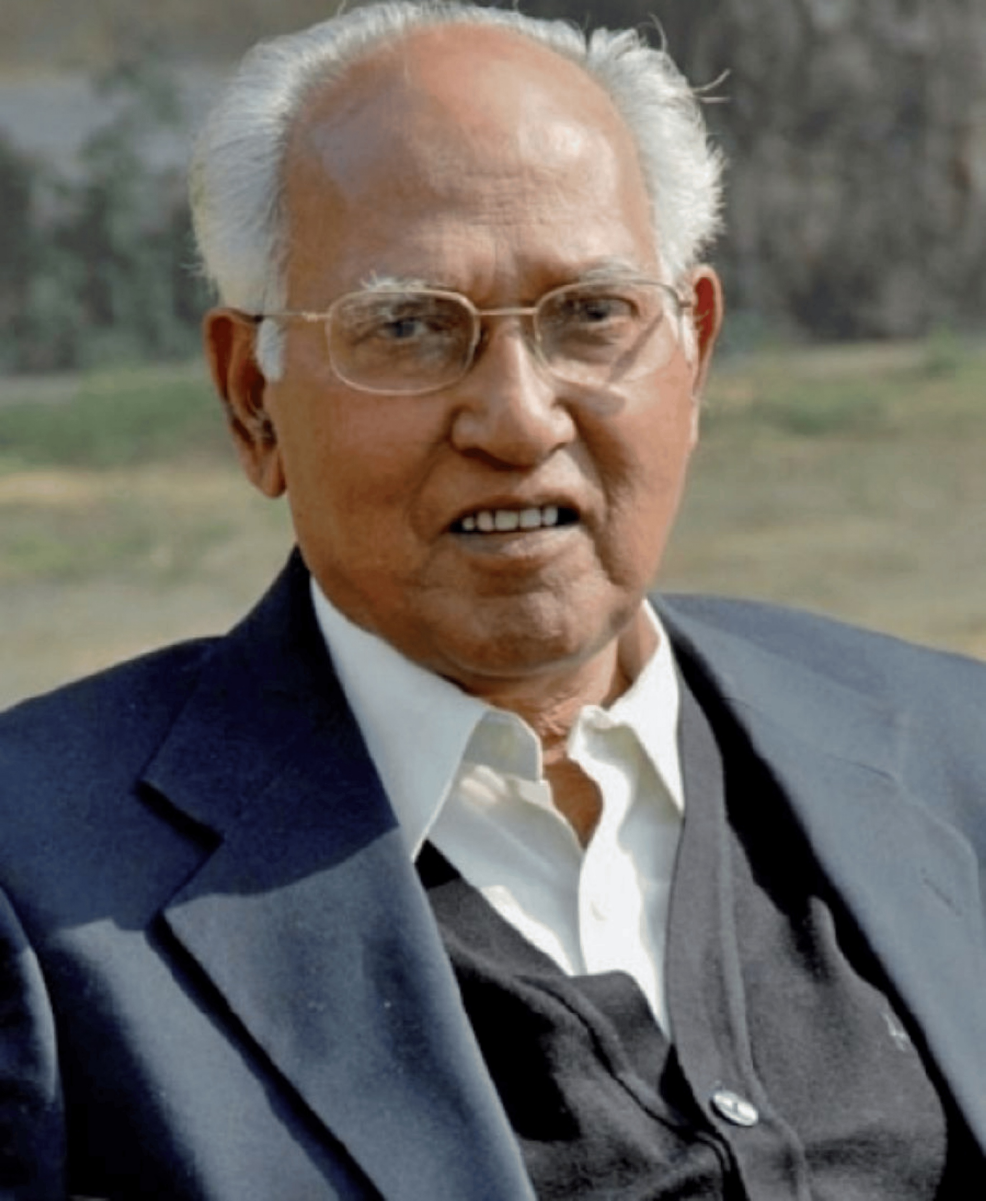
Dr. Pramod Karan Sethi (1927–2008) This photograph has been obtained from Bhargava (2008) [4], which is an open-access article distributed under the terms of the Creative Commons Attribution License, which permits unrestricted use, distribution, and reproduction in any medium, provided the original work is properly cited.

Dr. Pramod Karan Sethi, born on 28th November 1927 is known for the creation of the famous "Jaipur Foot". He left for heavenly abode on 6th January 2008 [1]. He was born in Banaras [2], to Dr. N.K. Sethi, a professor of physics at Banaras Hindu University (BHU). He and Pandit Ram Chandra Sharma, co-developed the "Jaipur Foot," in 1969 [1]. He gained international recognition for his creation of the "Jaipur Foot," a low-cost, flexible, durable, and waterproof prosthetic foot composed of readily available materials, which made it easier for amputees to walk on uneven terrain, sit cross-legged, and squat [3]. Dr. P. K. Sethi’s (Figure 1) groundbreaking work earned him numerous accolades.

## Review

Early life and education

Dr. Sethi received his initial education in Agra and completed his MBBS from Sarojini Naidu Medical College in Agra in 1949. In 1952, he completed MS general surgery [3]. He passed the MBBS examination with the highest marks in seven subjects. In 1954, he graduated with an FRCS (Fellow of Royal College of Surgeons) degree from Edinburgh, UK. After returning to India, Dr Sethi was appointed as a surgery lecturer at Jaipur’s Sawai Man Singh Medical College, where he established the exclusive department for orthopaedics and rehabilitation unit and served there for the next 28 years until his retirement in 1982. He saw plenty of amputees, primarily among young individuals who had lost their legs in road accidents [5]. He established the physiotherapy and occupational therapy departments in response to the necessity for his patients' rehabilitation following the conclusion of their medical care [6]. After retirement, he continued his research work and professional career at Santokba Durlabhji Memorial Hospital, a local hospital [2].

The Solid Ankle, Cushion Heel (SACH) foot

The earliest prototypes of the current SACH Foot were created in 1957 by J. Foort and C.W. Radcliffe of the Prosthetic Devices Research Project, Institute of Engineering Research, University of California, Berkeley. Since wearing shoes was required, all the nations with a barefoot culture rejected it. Most of these were agricultural nations, with workers tiling water-filled fields, an environment that was neither appropriate nor conducive to the employment of this foot. The majority of rural people in all of these countries walked or worked on uneven ground, which interfered with their floor-sitting lifestyle and did not quite fit well with this. The amputee's ability to squat or sit cross-legged was rendered impossible by the wooden keel in the SACH Foot, which inhibited any imitation of the ankle or sub-tarsal movements of inversion and eversion. Its attachment to the wooden shank constituted a bigger issue. The wood was hollowed out and moulded to resemble the stump for the shank. Because wood was hard to come by, it took time and was expensive and heavy [7]. Hence, the Western limbs that were readily accessible were too rigid and costly to give people the desired range of motion [8].

Innovation rooted in necessity: the genesis of the Jaipur Foot

Dr. Sethi aimed to offer amputees and polio victims suitable and affordable equipment. The closest artificial limb centre was located in distant Pune or Mumbai, accessible exclusively to the wealthy. To produce some of the aids locally, Sethi set up a workshop on the hospital premises. The Army Limb Centre in Pune created a foreign foot that was heavy, and stiff and required shoe coverage. As a result, many bought them but quickly stopped using them [9]. Like the majority of orthopaedic surgeons of his generation, Sethi was first trained as a general surgeon. In 1958, the principal of Sawai Man Singh Medical College urged Sethi to establish the orthopaedic surgery department in the local medical institution. Despite his initial reluctance, he quickly came to understand the advantages and disadvantages of contemporary orthopaedics in comparison to the resources and requirements of developing nations. It was an eye-opening experience for him to develop the low-cost, maintenance-free Jaipur Foot utilising locally accessible materials, local artisans' abilities, and technology. He observed that the main causes of orthopaedic issues in developing nations include deformities, infections, untreated injuries, and limited access to medical facilities, all of which are consequences of poverty and illiteracy. He quickly concluded that the majority of the pricy, high-tech orthopaedic problem solutions were only appropriate for a tiny portion of the world's population who could afford them. The fact that Dr. Sethi was not constrained by conventional thinking and could think "outside the box" let him recognise that his lack of formal training as an orthopaedic surgeon was a benefit rather than a drawback [2].

The Jaipur Foot

Pandit Ram Chandra Sharma, a local craftsman, once contacted Dr. Sethi with the notion of designing a new prosthesis. Together, they created a novel prosthesis intended for those who have undergone below-knee amputations [5]. A significant development occurred when the skilled craftsman, Pandit Sharma, created a die for a realistic-looking foot that could be easily cast in any size using a traditional sand casting technique [10]. The two proceeded by trial and error until they reached a hinged wooden ankle encircled by a rubberised foot [8]. Eventually, the adaptable, firm-gripping "Jaipur Foot" was produced in three shades - light, medium, and dark brown - and a split next to the big toe was made possible to accommodate sandal wear [10]. The resultant foot, commonly referred to as the "Jaipur Foot" globally, can be constructed for an individual in 45 minutes using plaster of Paris and lasts for five years. It is made of three different kinds of rubber: a microcellular rubber for the heel that has been sliced to allow for joint-like movement, a material piece for the metatarsals, and a shell filled with sponge rubber [8].

Only about 60 Jaipur foots and limbs were produced in the first 5 years [11]. It’s popularity increased during the Afghan war when Russian forces placed countless landmines. Millions of amputees around the world have been fitted for it since then, many of them were injured by landmines and other ordnance. Sethi dispatched associates from Jaipur to transport the technology to Bangladesh, Vietnam, Cambodia, and other countries [8].

The foot's original design featured a high ankle block of wood that made it easier to attach the prosthesis to the aluminium shank using wooden screws. The block was made of layers of sponge rubber that were glued together to mimic the movement of the ankle, subtalar, and midtarsal joints in a normal way. Originally, a wooden wedge with string lining for plantar support served as a duplicate of the tarsus and metatarsals. Every block was encased in a durable rubber shell that was vulcanised after being coated with natural rubber. Subsequent variants have a firm rubber block or layers of sponge rubber in place of the forefoot. It has been demonstrated in the lab that the sponge rubber components permitted dorsiflexion, plantar flexion, supination, pronation, internal and external rotation and that they also produced a more natural gait that was more in line with the functions of the regular foot [12].

Dr. Sethi was told to refer to the foot as "Sethi's Foot" during his first technical presentation. Refusing, he called it the "Jaipur Foot", after the city that served as his *karmabhoomi *(place of work) for the entirety of his existence. Despite having the potential to make millions, Sethi chose not to patent his idea. He was aware that anyone with basic fabrication skills could create this foot with a local craftsman in any community, so let everyone who could make it, produce it [2]. In 34 years time, from 1975 till 2009, 3.6 lac (360,000) people benefitted from the Jaipur Foot [13].

The major drawback of the Jaipur Foot is that it is heavy, and there is a lack of standardization of raw materials and fabrication process, due to which quality control cannot be maintained effectively [14].

Dr. Sethi's revolutionary callipers for polio-affected individuals

Dr. Sethi had other research interests and inventions. Since the 1950s, he tried to offer appropriate callipers to polio victims. The primary cause of immobility and deformities in India was polio, and similar to Western prosthetic limbs, Indian patients were not a good fit for the heavy, painful, and costly callipers used for the disease [2]. Under the guidance of Dr. Sethi, light, inexpensive callipers (braces) were fabricated by skilled but unlettered artisans who were proud to be socially useful [10]. Dr. Sethi worked with the National Chemical Laboratory in Pune and the Indian Institute of Technology in Mumbai to create callipers [2] of polyurethane for polio-affected individuals [5]. Dr. Sethi, along with IIT Mumbai’s professor S.C. Lakkad, worked on fabricating lightweight callipers using carbon fibre composites. In 1994, this was standardized by the Defense Research and Development Organization (DRDO) and Nizam Institute of Medical Sciences, Hyderabad. Nine thousand polio patients had benefited all over the country with this technology till 2008 [4]. He also set up the Rehabilitation Aids and Limb Fitting Centre (RALFC) in 1985, which aims to create and develop easy-to-use, low-cost, and lightweight rehabilitation aids that attach seamlessly to patients [15].

Story of Sudha Chandran

Born on September 27, 1965, Sudha Chandran is an Indian actress and Bharatanatyam dancer who works in Indian films and television shows. She had a right leg injury in a car accident in 1981 while returning from Madras, close to Tiruchirapalli, Tamil Nadu. Her parents decided to have her right leg amputated after it developed gangrene [16]. Six months after the amputation, Sudha came across an article in a magazine about Dr. Sethi, a Jaipur-based artificial limb specialist who was honoured with the Ramon Magsaysay Award [17]. These legs had such great capabilities that a man wearing them could labour on a farm and even climb trees [18]. Sudha sent him a letter.

Dr. Sethi did all within his power to create a prosthetic foot that would meet Ms. Chandran's dance needs [17]. Taking on the task as a challenge, Dr. Sethi had an aluminium foot made that was incredibly light. To facilitate easy leg rotation, a configuration was devised. He took his time studying and evaluating her legs' varied movements while she danced. He made arrangements for a new leg while keeping in mind the dance's requirements.

Ms. Chandran was scheduled to perform in a dance performance at the Mumbai "South India Welfare Society" hall on January 28, 1984, with another dancer named Preeti. This period was quite challenging for her. However, the programme was considered very successful [18]. She reportedly "just forgot that her leg was artificial and started dancing swiftly" as soon as she appeared on stage. Not one person blinked as they continued to stare at her. The sound of applause filled the hall as the performance concluded [6]. Reviewers of dance praised the show. There were many descriptions, compliments, and images in newspapers and magazines. Sudha had suddenly shot to fame.

With the help of the Jaipur Foot design, Ms. Chandran overcame despondency and started her subsequent career. In addition to praising her, a well-known Telugu film producer chose to make a movie based on her life narrative [18]. She even performed a rigorous dance sequence in the movie “Nache Mayuri” [19].

Awards and achievements

The *Time *magazine praised Jaipur Foot as one of the best innovations of the 20th century [7]. Dr. Sethi received widespread praise for his work on the "Jaipur Foot" when he initially presented it at the Association of Surgeons of India's annual conference in Bangalore and the British Orthopaedic Association meeting in Oxford that same year. Dr. Sethi was rewarded with numerous prestigious scientific awards like the Guinness Award for Scientific Achievement, the Knud Jensen Medal, the R. D. Birla Award for Outstanding Medical Research, and the Western India Orthopaedic Society Gold Medal. The title of eminent medical man was bestowed upon him, for which he was awarded the Dr. B. C. Roy National Award in 1989. Along with, all these honours, he was invited to present guest lectures both within the country and abroad to present his acclaimed speeches. He was bequeathed the Lifetime Achievement Award by the Indian Orthopaedic Association in 2004. He was conferred the most prestigious Padma Shri in 1981 by the Indian government [3]. He also received the Ramon Magsaysay Award, also known as the “Asian Nobel Prize” [11].

Impact on rehabilitation and community development

The Jaipur Foot proved to be a fairly priced, suitable, and elegant alternative to the Western leg design that was available at that time. Thus, many amputees from the developing and underdeveloped countries now lead a drastically changed, significant lives [20]. Dr. Sethi’s will to change the way people with disabilities live and his sheer observation of the discomfort and problems the amputees experience in India, especially those who come from low-income families, helped him create this inventive device, which was low-priced and was made of basic components that was accessible to the general population [21]. There have been no reports of any falls or sudden breaks in the device that could have harmed the patients. This proves that it is a firm, secure rehabilitation device for people whose limbs are amputated. The Jaipur Foot has proven that during exercises for rehabilitation and while sitting and squatting or sitting cross-legged it doesn’t affect the area of injury nor does it change the degree of where the prosthetic feet are placed [22].

A comparison study by Prof. K. Adalarasu in 2011 on Jaipur Foot, Madras Foot, and SACH (Table 1) showed that the Jaipur Foot prevailed over the others in aesthetics, sociocultural acceptance, exhaustion rate, composition, and performance [23].

**Table 1:** This table outlines the key performance and feedback parameters for Jaipur, Madras and SACH foot [23].

Feature	Jaipur Foot	Madras Foot	SACH Foot
Shock Absorption	Good	Least	Best
Abrasion (Volume Loss)	Acceptable	High	Acceptable
Life Span	> 3 yrs	2 -2.5 yrs	2.5 -3 yrs
Light Weight Comfort	Excellent	Least	Good
Comfortable Walking	Excellent	Least	Excellent
Balancing with Prosthesis	Good	Least	Good
Limping	Least	High	Satisfactory
Ease in Walking Barefoot	Good	Least	Good
Occupation Regain (Retrieve Rate)	77.9%	Not specified	Not specified
Training Satisfaction	Good	Good	Good

A cross-sectional pilot study conducted by Dr. Rashmi Yeradkar and Dr. Shailaja Jaywant in Mumbai, India, in 2020 concluded that there is a positive correlation between prosthesis satisfaction and functional mobility. In lower limb amputees, this influences early return to work [24]. A comparison study of the Jaipur Foot, Seattle, and SACH by A. P. Arya et al. in 1995 showed that the SACH foot had better shock absorption capacity when compared with the other two feet, while the Jaipur Foot allowed a more natural gait and was closer in performance to the normal foot [25].

## Conclusions

Dr. Pramod Karan Sethi transformed the lives of amputees in developing and underdeveloped countries by developing the Jaipur Foot. It is a cheap, accessible alternative and culturally appropriate compared to the western prosthesis of that time. He utilised regional resources and artisans to create the Jaipur Foot, which helped amputees regain their freedom and movement. The Jaipur foot demonstrates the philosophy of using locally available materials and resources to find solutions to challenging problems in the developing world. Jaipur Foot was the most cost-effective prosthetic technology of its time in the world. The increase in locomotor disability all over the world makes this technology more relevant due to its simplicity and low cost. Dr. Sethi refused to patent the design, prioritized humanitarian values and ensured widespread and easy access to Jaipur Foot. His work set a new standard in rehabilitation and healthcare innovation and left a legacy that will continue to inspire advancements in prosthetics globally.
